# The nursing legacy of the Korea Sisters

**DOI:** 10.1002/nop2.117

**Published:** 2017-12-27

**Authors:** Jan‐Thore Lockertsen, Åshild Fause

**Affiliations:** ^1^ UiT – The Norwegian Arctic University Tromso Norway

**Keywords:** education, Korean War, theatre nurses, wartime nursing

## Abstract

**Aim:**

During the Korean War (1950–1953), the Norwegian government sent a Mobile Army Surgical Hospital (MASH) to support the efforts of the United Nations (UN) Army. During the war, 111 Norwegian nurses served in seven contingents, each 6 month, at the Norwegian Field Hospital in Korea. The nurses were nicknamed “The Korea Sisters”. The aim of this study is to explore the impact and influence of their wartime nursing on Norwegian post‐Korean‐War nursing.

**Design:**

Qualitative.

**Methods:**

The study uses several historical research approaches. Interview, archival search, search in nursing periodicals, contemporary magazines and nursing text books.

**Result:**

The nursing legacy of The Korea Sisters can be found in changes in general nursing, uniform education of theatre nurses, uniform education of anaesthetist nurses and in humanitarian work.

## 1 INTRODUCTION

Modern nursing is unbreakable linked to Florence Nightingale and her well documented organizing of nursing in 1854–1855, during the Crimean War (1853–1856). Through improvement in sanitary conditions and carefully nursing of fever and diarrhoea at the barrack hospitals in Scutari, the mortality rate among causalities fell considerably (Elstad, Glasdam, & Bydam, 2008). Nurses knowledge and skills are tested in wartime and emergencies. Competence tried and practice expanded and changed (Helmstadter, 2015).

Wartime nursing is a growing research field among nurse historians as well as historians with interest in gender and war. Several scholarly works have been published both about specific wars and topics, as well as anthologies about nursing in wartime. For one war, however, the material is scarce. The Korean War. It is claimed that nursing in war is a catalyst for change (Brunk, 1997). Medical advancement resulting from The Korean War, have been identified and discussed (Baker, 2012), but what about nursing?

During the Korean War Norway established and ran a field hospital in Korea. Norway did not have a nursing corps or nurses trained for military service. A total of 111 civilian Norwegian nurses served at the Norwegian Mobile Army Surgical Hospital, NORMASH. The purpose of this article is to address the impact of their nursing experiences on Norwegian post‐Korean‐War nursing.

The Korean War started 25 June 1950 when North Korea attacked South Korea. The United Nations condemned North Korea and responded to the aggression with a peace enforcing UN Army led by the USA. The hostilities ended with an armistice 27 July 1953. One and a half million soldiers and civilians are believed to have been killed and another two and a half million mutilated or wounded.

From July 1951–November 1954 Norway participated in the UN Army with a MASH serving 15–30 km behind the combat zone. The MASHs were designed to have a capacity of 60 in‐bed patients and four operation tables. The purpose was to give war causalities quick and definitive treatment. Norway did not have an army nurse corps and depended on civilian nurses to staff the field hospital. The nurses served in seven contingents, each 6 month. A total of 9600 operations were performed at NORMASH. All in all, over 90,000 patients were received and treated (Dale, 1955). The Korean War is the largest war zone mission ever carried out by The Armed Forces Medical Services of Norway (Malm, 1951).

The nurses were nicknamed “The Korea Sisters” (Hetty & Rønnaug, 1951). During the war, Korea Sisters were interviewed in both newspapers (Aftenposten, 1952) and parish news (Hammarøy, 1953) and their humanitarian engagement in Korea was well known. After the war, the nurses faded from the public eye and their efforts are at present almost forgotten. They have not been a topic for Norwegian nurse historians (Fause & Micaelsen, 2001; Mathisen, 2006; Wyller, 1990). But among old nurses, they are still remembered and sometimes mentioned, as when one nurse said: “Sister Ragnhild [Strand] was a skilled theatre nurse, she had been in Korea” (Bockelie, 2009).

In this article, I raise this question: Whether and in what way “The Korea Sisters” experiences in Korea had any impact on Norwegian nursing, after the war? And, did the Korea Sisters humanitarian engagement in Korean, end when the Norwegian Field Hospital was terminated in October 1954?

I will highlight the nurses’ educational background and their preparedness for nursing in a war zone in a country far away from Norway, while investigating outcome of their wartime nursing. Did their wartime nursing act as a catalyst for identifiable changes in education and training of Norwegian nurses?

## 2 METHODS

Nursing history uses several methods in researching historical questions. In this study, archival search, memoir books, contemporary journals, nursing periodicals, nursing text books and oral history, have been used to identify participating nurses and research the impact of their practice on Norwegian post‐Korean War nursing (Lewenson and Herrmann (2008); Duffin, 2010).

Two memoir books have been written about the Norwegian Field Hospital and Norwegian efforts during the Korean War (Bakke, 2010; Pedersen, 1991). These books were written by soldiers and not by nurses (an excerpt from Unni Foss’ diary is included in Pedersen (1991:49–52). According to Gerd Semb, Foss's diary was given to a museum, but I have not been able to locate it—from interview with Gerd Semb, conducted by Jan‐Thore Lockertsen, (Lørenskog, 20 September 2011). Andresen, Ruth's chapter “Norsk feltsykehus i Korea”, in: *Fra norsk sanitets historie: sjefssøster forteller om kvinners innsats i militær sykepleie*, OSLO: NKS‐forlaget, 1986:103–108, is an overview chapter about the service in Korea). The two books include the names of all 698 personnel at NORMASH and names all participating nurses. In 1960, an encyclopaedia, *Norske sykepleiere*, was published, listing most of the registered nurses in Norway and their education and practice (Straume, 1960). This encyclopaedia lists nearly every Korea Sister, as well as their practice before and the first decade after the Korean War. To identify changes in practice related to the service during the Korean War, the Norwegian Nursing Association's (NNA) periodical *Sykepleien* has been searched for topics related to theatre‐ and anaesthetist nursing, Korea and the expression Korea Sister for the period 1951–1980.

Oral history is considered the most effective way to collect testimonies from eyewitnesses (Thompson, 2000; Boschma, Scaia, Bonifacio, & Roberts, 2008). The Korean War ended over 60 years ago. Very few of the nurses are still alive. Six nurses and one surgeon, all in their nineties, have been identified and interviewed for this project. They have all been interviewed in their own homes in an open form. Through their stories, their own and other nurses’ practices have been identified.

These sources have inspired by Lindseth and Norberg (2004), been condensed and interpreted into these themes: Pre‐Korean War: educational background. Post‐Korean War: general nursing; establishing a uniform theatre nurse education; theatre nursing evolves into two disciplines and humanitarian aid.

Archive search at the National Archives of Norway (Riksarkivet), Oslo and Anno Museum, Hamar, with deacon and nurse Gotfred Rekkebo's (1911–1993) private archives, have been conducted. Rekkebo served in contingent one in 1951 and did methodical produce scrapbooks before and after The Korean War. Those two archives are not holding any material related to nursing after The Korean War. They have still been useful for sure identification of names of the nurses. Obituaries and online recourses have been searched, helping illuminating nursing practices relevant for this project. The project has been ethical approved by NSD—Norwegian Centre for Research Data.

### 2.1 The professional background of the nurses

**Figure 1:**
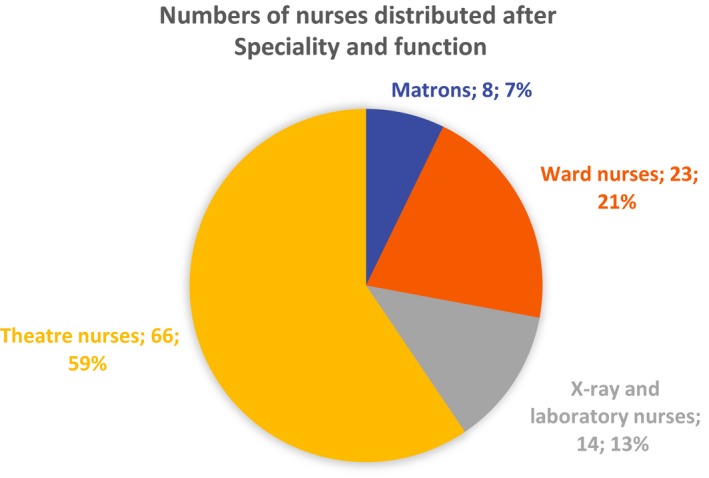
This diagram illustrates the number of nurses distributed according to speciality and function at NORMASH

NORMASH was a surgical hospital. The most demanded were nurses trained as theatre nurses. Criterions for selection of nurses was education and training and newer practice from surgical hospital. Other criterions were language knowledge and behaviour (Andresen, 1951). Their staffing plan was: one matron (head nurse) for the hospital and one head nurse for the operating theatre; seven theatre nurses cross‐trained as nurse anaesthetists; two x‐ray nurses; one laboratory nurses. Finally, there were six positions for ward nurses. This diagram illustrates the number of nurses distributed according to speciality and function at NORMASH (Figure 1).

Most of the nurses were trained as theatre nurses. Sixty‐six nurses, 59%, worked in the operating theatre. Twenty‐three registered nurses filled positions as ward nurses. Twenty‐two deacons served at NORMASH together with the registered nurses in five contingents. Deacons were trained male nurses, but their education was mainly directed towards congregational and social work. Nurse training was mostly seen as a way into administration and social work (Stave, 1990:269). The service at the x‐ray department laid the foundation for further surgical intervention. Positions as x‐ray nurses and laboratory nurses were never filled according to the staffing plan.

The Korea Sisters were all educated in a 3‐year programme during the years between 1930–1947. All of them had therefore experienced the Second World War and the occupation of Norway by Germany (1940–1945), either as practising nurses or as pupils. Theatre nurses’ training consisted of a 1‐year apprenticeships at municipal hospitals or 2‐year apprenticeships at Red Cross hospitals after nurse education (Ordrop, 1953:13). The average age when serving in Korea was 34 years.

Considering the education and training of the two senior nurses in contingent one, reveals interesting information about the level of education and competence among the Korea Sisters. Matron (head nurse) Rønnaug Wüller had, as part of her training as theatre nurse, secondments in Denmark in 1939–1940 and had courses in hospital administration from both Denmark and England. Before her service in Korea, she had held positions as head nurse in both medical and surgical departments. Also head nurse operating theatre, Orlaug Sofie Haarvik had further education in administration and teaching. In addition, she was educated as a missionary and had been head nurse in a Norwegian hospital in central China between 1946–1950. Analyses of the background of the other Korea Sisters support the impression that the nurses were skilled and experienced nurses (Straume, 1960).

Through membership in the International Council of Nurses, NNA was oriented towards international cooperation between nurses. Members were encouraged to travel abroad for secondments and further education (Hvalvik, 2005). Secondments abroad to study surgical techniques and new methods, as well as design of operation theatres, were encouraged and became popular. Twenty‐four of the theatre nurses at NORMASH are reported to have studied or had secondments in hospitals abroad, mostly in operation theatres (Isaksen, 2012; Straume, 1960). Some nurses also used the opportunity to visit USA for to study nursing after finishing their service at NORMASH (Andresen, 1951).

In 1925, NNA founded a school for further education in nursing, where courses in hospital administration and administration of operation theatres, teaching and public health nursing and social work were taught (Melby, 2000). Ten Korea Sisters had further education from NNAs school in addition to theatre nurse training, eight in hospital administration and administration of operating theatres and two in social work. Four of the theatre nurses had also worked for the WHO as vaccinators in programmes for preventing tuberculosis. Two were midwives. In addition to the theatre nurses, seven of the other nurses were trained as specialists in laboratory nursing and seven in x‐ray nursing (Straume, 1960).

In 1948, theatre nurses in Oslo formed and organized an interest groups that sought to influence their working conditions. The primary goal of the theatre nurses’ interest group was to form a uniformed education in theatre nursing. Interest groups like the theatre nurses, Norsk Sykepleierforbunds Landsorganisasjon av operasjonssykepleiere (NSFLOS), were very influential in the NNA when it came to professional issues (Lund, 2012). Nineteen later Korea Sisters were active in NSFLOS from the very start. One of them, Berit Røe, designed the emblem still used by NSFLOS today (Høiland, 1996).

In October 1954, NORMASH stopped receiving new patients and in November 1954 it closed. The Korea Sisters had performed war surgery and trauma surgery like no countrymen before them. They taught soldiers, chauffeurs, cooks and others in basic nursing techniques and supervised them during rushes when all hands were needed in the operation theatre to take care of causalities and had experienced wartime nursing in a way unknown to fellow nurses (Granå, 2004). After the armistice, NORMASH came to function as a civilian hospital as much as a military hospital. The Korea Sisters also started training Koreans in nursing as part of the reconstruction of Korea.

The Korea Sisters was as a group nurse's eager to form and develop professional standards for nursing. Before the Korean War, they had sought both training and education international. National, they were engaged in developing the training of theatre nurses to a national uniform education. Had the nurse's newly acquired wartime nursing practices any impact on Norwegian nursing after the Korean War? Was their hardship only an intermezzo in their lives and Norwegian nursing? Did they just go on back to Norwegian operating theatres or other branches of nursing, without using their experiences for the benefit of Norwegian nursing?

## 3 THE KOREA SISTERS IMPACT OF NURSING PRACTICE IN NORWEGIAN NURSING

### 3.1 General nursing

The MASH concept, with a moveable hospital close to the battlefield, was first tried out in the Korean War. Rapid evacuation, closeness to the battlefront and the high number of causalities provided much clinical practice in trauma reception and trauma surgery. Infection control with antibiotics was routinely used on all traumas, which lowered the mortality rate. But antibiotics could not substitute for nursing skills in wound treatment and infection control. The nurses experienced that mechanical debridement of wounds and delayed primary closure was necessary (Hartvigsen, 1954; Paus,^1954^). Similar advantages of delayed closure were later experienced by Australian field hospitals in 1967 during the Vietnam War (Biedermann, 2002:337).

Mobile facilities like MASH demonstrated the value of early definite care, which has since been adopted in civilian health care and disaster preparedness (Gawronski, 2015). In Norway, Korea Sisters were a great part of introducing this new knowledge into general nursing. Several of them became matrons and head nurses in hospitals and in charge of the nursing services at their hospitals or wards. Matrons were also in charge for education and training of nurses at their respective hospitals (Melby, 2000).

Still, most of the Korea Sisters were active as theatre nurses or nurse anaesthetists. Many of them were gainfully employed until the late 1970s. All nurse pupils and from 1976 students, in Norway, had long practice as a part of their education at the operation theatre at least until the 1980s. They met Korea Sisters or other nurses who had learnt much of their practice at NORMASH. Korea Sisters are also known to have given lecturers on military nursing to nurse pupils (Johansen, 2007) and rescue corps based on principles learnt from service in Korea (Karlsen, 2016). Up to the late 1990s, many students and nurses also used the handbook, “Prosedyrebok for sykepleiere”, drafted by Korea Sister Berit Røe et co (Røe & Martinsen, 1986).

Theatre nurses at NORMASH were deployed for no more than one term in Korea. Chief Nurse of the Army, also a Korea Sister, Ruth Andresen, wanted as many nurses as possible to have the experience in case similar situations arose in Norway. Many of the Korea Sisters joined the army reserve afterwards, ensuring that Norway had theatre nurses with first‐hand experiences in operating mobile hospitals and handling many patients needing surgical intervention in a war zone (Sykepleien, 1976).

One of the Korea Sisters, Hetty Henrichsen, advanced from assistant executive secretary to executive secretary and finally leader of the Norwegian Nursing Association between 1965–1967, the most influential position for a nurse in Norway (Lund, 2012). During her period as leader, NNA launched the textbook series “Lærebok for sykepleieskoler” in twelve volumes (Henrichsen, 1967).

### 3.2 Establishing a uniform theatre nurse education

The Korean War was not the first war where knowledge of Norwegian theatre nurses was sought. Already during the First Balkan War (Oct. 1912–May 1913), five theatre nurses were part of a Norwegian ambulance. They were selected for this mission because they were theatre nurses (Norsk Sykepleierske‐Forbund, 1912). Norwegian theatre nurses were also engaged in Finland during the Finnish Civil War (1917–1918) (Natvig, 1918) and in Finland again during the Winter War between Finland and the Soviet Union (1939–1940). Theatre nursing was recognized as a specialization, but there was no formal education in theatre nursing.

The preparation for the Norwegian Nursing Act of 1948 showed a consensus in Norway for establishing theatre nursing as a specialization building on 3‐year uniform education as a nurse (Ot.prp. nr. 31, 1948). But a curriculum with a programme in theatre nursing did not exist, only a training (Wyller, 1956). It consisted of 1 year of training or 2 years of internal training in an operation theatre (Ordrop, 1953:13). This was too haphazard for the theatre nurses themselves. The Korean War provides examples of up‐to‐date theatre nursing, but also the opposite can be seen.

There are records of assistance being provided without sterile gown and gloves at NORMASH. While this shows handiness, such practices were also outdated and could pose a potential risk of infection for patients. Without national guidelines and nursing instructors, old techniques and techniques not brought up to date with the newest knowledge might nevertheless end up being standard procedures at some hospitals. Establishing a national standard for nursing in operation theatres was a priority. Through their own profession group, NSFLOS, theatre nurses worked to establish a uniform education for theatre nurses.

Korea Sisters were active in NSFLOS effort to establish a uniform education for theatre nurses. In a letter dated 1959 from Hetty Henrichsen, then the executive secretary of NNA, to Korea Sister Kitty Tyskø, it is stated that status regarding the work with a theatre nurse education would be given at the forthcoming national congress (Henrichsen, 1959). A year later, Tyskø oversaw the development of a curriculum for theatre nursing training at the Hospital in Akershus (Hvoslef & Jørgensen, 1976).

Not all work in an operating theatre had to be done by nurses. The Korea Sisters themselves had good experiences with delegating theatre nurses’ tasks to assistants. Maintaining instruments, packing and sterilization were tasks they delegated to civilian Koreans and guard soldiers in their spare time—and with very good results, according to Kari Roll Klepstad (Klepstad, 2011). After retiring from NNA in 1967, Hetty Henrichsen became active in establishing an education for auxiliary nurses. Regarding the patient‐related work, the high nursing competence among the Korea Sisters has been taken to be one of the main factors for the good results achieved at NORMASH (Moe, 1953). The question about substituting theatre nurses with personnel with less education, like auxiliary nurses, in role of theatre nurses, was discussed, but never serious considered (Lockertsen, 2009). This was probably due to the good experiences with using only competent personnel at NORMASH.

The Korea Sisters contributed to various parts of the struggle to establish a uniform theatre nurse education. In Korea, they had shown what educated nurses could do in a crisis. Back home in Norway they contributed to daily work in operating theatres, some of them in senior positions as head nurses for the operating theatres, where they were responsible for the instruction of theatre nurses. Others, like nurse Berit Røe, had more formal training as teachers. Several hospitals developed curricula and employed teaching managers, making the education of theatre nurses more structured and formal.

With such efforts being made in many hospitals, it was only a question of time before NSFLOS's curricula became the start of education after national guidelines. By 1973, curricula from the theatre nurses were used from Bodø in the north of Norway to Arendal in the south (Gjendem, 1974). By 1975, all main hospitals in Norway educated theatre nurses following national guidelines that ensured a uniform programme of education.

### 3.3 Theatre nursing evolves into two disciplines

Norway got the first specialist in anaesthesiology in 1947 (Skagestad, 1999). Without anaesthesiologist, the standard procedure in Norway was that the surgeon had the responsibility for anaesthesia and a theatre nurse conducted anaesthesia. This solution was continued at NORMASH. It worked for injuries in the extremities and even in the abdomen. But it became very clear that war surgery, demanded more knowledge. For thoracic surgery, more expertise was needed. Neither the theatre nurses nor the surgeons at NORMASH had sufficient knowledge in anaesthesiology for surgical procedures on severely wounded patients with thoracic and chest traumas (Lind, 2015). From the second contingent on, eight of the ten practising anaesthesiologists in Norway, served at NORMASH, working closely with the theatre nurses forming standards for wartime anaesthetist nursing (Lind, 1953).

Theatre nurses were aware that their profession needed more specialized knowledge of anaesthesiology, than cross‐training in anaesthetist nursing. In the years between 1945–1950 travels to Denmark to attend courses or have secondments in anaesthetist nursing, was not uncommon. Among the Korea Sisters, titles like “theatre nurse and nurse anaesthetist” can be found and in the first few years after Korea, several Korea Sisters used only “nurse anaesthetist” (Straume, 1960). This might indicate that theatre nurses themselves defined it as a separate specialization and thereby started to divide theatre nursing into two specialities.

In the 1950s, only brief courses for nurse anaesthetist existed, in Norway. These courses were discontinued because cross‐trained theatre nurses were given too much responsibility and were in some hospitals expected to fill the position of a physician who had specialized in anaesthesiology (Lind, 1965). The anaesthesiologists were too few to serve all hospitals in Norway. As an example, Korea Sister Ragnhild Strand at Tromsø Hospital was responsible for training theatre nurses and cross‐training in anaesthesia from 1955–1965 with no anaesthesiologist at the hospital.

The Korea Sisters participated in the development of an education for nurse anaesthetists. Korea Sister Ruth Nordby was the first chairman and one of the founding members of the interest group for nurse anaesthetists in 1957. The primary goal for this group was establishing of a uniformed education for nurse anaesthetists (Hopen, Jansen, Engevik, & Olsen, 2013). At the Hospital in Akershus, Korea Sister Mary Jensen oversaw developing a curriculum and education programme for nurse anaesthetists (Hvoslef & Jørgensen, 1976). In 1965, the NNA approved Nurse Anaesthetist as a separate speciality, rather than a branch of theatre nursing. Still, most theatre nurses were cross‐trained in anaesthesia until a nationwide education programme following national guidelines was established in 1974–1975.

For decades, nurses had travelled abroad to seek secondments as part of their training. Other countries’ nursing knowledge became known to and studied by Norwegian nurses. The Korean War was a meeting point with nursing traditions of other countries. NORMASH was in many ways a whole hospital in secondment. Anaesthesiology was not properly covered by specialists and theatre nurses were unable to provide the anaesthesia needed. The Korean War accelerated the separation of anaesthetist nursing from theatre nursing and development of a formal education for nurse anaesthetists.

### 3.4 Humanitarian aid

The end of NORMASH was not the end of the Korea Sisters’ engagement on behalf of Korea. Already in 1951, it was preliminary decided that a joint Scandinavian hospital should be established. In 1956, Sweden, Norway, Denmark, The Republic of Korea and the UN agreed in establishing a medical centre for training health personnel (United Nations Korean Reconstruction Agency, 1956). On 2 October 1958, the National Medical Centre (NMC) in Seoul was opened and it was run by the three Scandinavian countries for 10 years before being transferred to the Koreans in 1968.

During that decade, 216 Scandinavian nurses served at the NMC and nine of the 58 Norwegian nurses had previously worked at NORMASH (Bakke, 2010). The impact of Scandinavian nursing is recognized by nurses in Korea: “Though not calculated by a scientific measure, it is presumed that the Danish, Norwegian and Swedish influence on nursing profession in Korea is immense” (Halm, Kim, Lee, & Park, 1998). NMC is still a working hospital in Seoul, Korea. The nurses who contributed to the reconstruction of South Korean health care by serving as head nurses and nursing instructors at NMC must, in a broader sense, be named Korea Sisters. Through cooperation with nurses from Denmark and Sweden they gained experiences that they later brought home to Norway.

The NMC was a humanitarian project with the purpose of contributing to reconstructing Korean health care after a war. Inga Årdalsbakke served both at NORMASH and at NMC. She considered NMC to be a continuation of NORMASH (Årdalsbakke, 2013). Via the Korean War the NMC provided the ignition for Norwegian nurses to participate in organized humanitarian work on a larger scale than missionary nursing. In fact, Norwegian studies indicate that nurses participating in missions in war zones after the Korean War primarily view themselves as participating in humanitarian aid (Tjoflåt, 1996). For Norway as a nation, the NMC is recond as a part of the start of establishing Norway as a humanitarian superpower (Berg, 2016:143).

## 4 CONCLUSION

Civilian nurses participated in this first Norwegian foreign war mission after the Second World War. A closer look at the professional background of the nurses indicates that they were highly competent regarding serving in a field hospital in a war. The success of NORMASH as a hospital in a war indicates the same. Wartime nursing at NORMASH can most probably be attributable as the catalyst for identifiable changes in Norwegian nursing and education of nurses. The nurses’ impact on Norwegian nursing and education of nurses, may be identified in four fields:

First: The Korea Sisters’ clinical practice and surgical techniques were transferred to Norwegian nursing through daily work and lectures. Principles of treatment learnt in Korea were probably introduced into Norwegian nursing before they were introduced to nursing in other countries since nurses went back to work at civilian hospitals.
Second: Norwegian theatre nursing proved to be important practice for wartime nursing in Korea. Still, the nurses themselves realized that 1 or 2 years of internal training at a hospital's operation theatre was insufficient. They wanted a nationwide, uniform education with an authorization. Through theatre nurse's own profession group, the Korea Sisters also took part in drawing up a curriculum and working out a nationwide uniform education for theatre nurses. This programme became a reality in Norway in 1974–1975. The Korean War had shown that well‐educated theatre nurses were essential in the operating team. Their experiences also showed that tasks not related to patients but rather to logistics, such as preparation and sterilization of surgical instruments, could be delegated to non‐nurses.
Third: Nurse anaesthesia was performed by theatre nurses cross‐trained in anaesthesia and for some nurses, additional short course. The service in Korea revealed weaknesses in this area of nursing. Complicated cases required both anaesthesiologists and educated nurse anaesthetists. Theatre nurses with an interest in anaesthetist nursing were, after Korea, involved in dividing theatre nursing into two disciplines and establishing a nurse anaesthetist education in Norway.
Fourth: Norwegian nursing during the Korean War led to active participation in reconstruction and rebuilding of Korean health care. Sweden, Denmark and Norway continued their non‐combatant roles with the establishment of the National Medical Centre, with the purpose of educating nurses and physicians. Still more research is needed.

## CONFLICT OF INTEREST

There is no conflict of interest to be declared by the authors of The Nursing Legacy of the Korea Sisters.
